# Social drift of cardiovascular disease risk factors in Africans from the North West Province of South Africa: the PURE study

**DOI:** 10.5830/CVJA-2012-018

**Published:** 2012-08

**Authors:** PT Pisa, R Behanan, HH Vorster, A Kruger

**Affiliations:** Centre of Excellence for Nutrition (CEN), North-West University, Potchefstroom, South Africa; Centre of Excellence for Nutrition (CEN), North-West University, Potchefstroom, South Africa; Centre of Excellence for Nutrition (CEN), North-West University, Potchefstroom, South Africa; Africa Unit for Transdisciplinary Health Research (AUTHeR), North-West University, Potchefstroom, South Africa

**Keywords:** CVD risk factors, social drift phenomenon, socio-economic status, dietary intakes, PURE baseline study, North West Province, South Africa, serum lipids

## Abstract

**Objective:**

This study examined whether the association between socio-economic status (SES) and cardiovascular disease (CVD) risk factors in black South Africans from the North West Province had shifted from the more affluent groups with higher SES to the less affluent, lower SES groups over a period of nine years.

**Method:**

Cross-sectional baseline data of 2 010 urban and rural subjects (35 years and older) participating in the Prospective Urban and Rural (PURE) study and collected in 2005 were analysed to examine the relationship of level of education, employment and urban or rural residence with dietary intakes and other CVD risk factors. These relationships were compared to those found nine years earlier in the Transition and Health during the Urbanisation of South Africans (THUSA) study conducted in the same area.

**Results:**

The results showed that urban women had higher body mass index (BMI), serum triglyceride and fasting glucose levels compared to rural women and that both urban men and women had higher blood pressures and followed a more Westernised diet. However, rural men and women had higher plasma fibrinogen levels. The more highly educated subjects (which included both urban and rural subjects) were younger than those with no or only primary school education. Few of the risk factors differed significantly between education groups, except that more highly educated men and women had lower BMIs, and women had lower blood pressure and triglyceride levels. These women also followed a more prudent diet than those with only primary school education. Employed men and women had higher BMIs, higher energy intakes but lower plasma fibrinogen levels, and employed women had lower triglyceride levels. No significant differences in total serum cholesterol values were observed.

**Conclusion:**

These results suggest a drift of CVD risk factors from groups with higher SES to groups with a lower SES from 1996 to 2005, indicating that interventions to prevent CVD should also be targeted at Africans living in rural areas, those with low educational levels, and the unemployed.

## Abstract

The escalating global burden of cardiovascular disease (CVD) is related to the rapid health transition that developing countries are experiencing.^1^ Adoption of high-energy, high-fat, Westernised diets and diminished physical activity contribute to the accelerating epidemic of CVD.^2^ South Africa, an emerging economy, is currently undergoing a health transition, characterised by the triple burden of disease consisting of a high prevalence of under-nutrition-related infectious diseases, the emergence of non-communicable chronic diseases (NCDs), and the human immunodeficiency virus/acquired immune deficiency syndrome (HIV/AIDS) pandemic.^3^

The rate of urbanisation in the North West province of South Africa has dramatically increased, characterised by high rates of rural-to-urban migration, increased numbers of industrial companies, improved economic activity and an increased population in urban settings. Concurrently these economic changes have led to changes in the quality of food intake, from the traditional prudent dietary patterns and nutrient intake to modern, imprudent fast foods, which seem to play a major role in increasing the rate of NCDs.

The THUSA study, conducted from 1996 to 1998 in the North West Province of South Africa, indicated that Africans with a higher socio-economic status (SES) had higher risk factors for CVD than those with a lower SES.^4^ Many studies have shown that in developing countries, during the early phases of the health transition, the NCD/CVD burden is usually higher in the higher SES class.^2^,^5^,^6^ In developed countries, the burden has shifted to the poor.^7^-^11^ This phenomenon of a shift of the burden of CVD in a population from the rich to the poor can be seen as a social drift in CVD risk.

However, some studies found that the usual pattern is not always followed. In certain developing countries, higher CVD risk factors were seen in lower SES groups.^12^-^15^ A study from a developed country^16^ showed that higher CVD risk factors were found in higher SES groups. These studies illustrate a dynamic drift of the burden of CVD risk factors between groups of different SES within countries.

As mentioned above, the health transition in developing countries is closely related to the transition from prudent, low-energy, low-fat diets traditionally followed in rural areas to the more Westernised high-energy, high-fat diets^3^ followed in urban areas. There is convincing evidence that this Westernised dietary pattern increases the risk of CVD and other NCDs through a variety of mechanisms.^17^

Diet can, therefore, be seen as a risk factor for CVD and dietary recommendations create the cornerstone in prevention of CVD.^17^ To be effective and successful, any dietary intervention programme should be focused, using dietary messages, educational material and nutritional advice targeted at specific groups.^18^ It is therefore important for prevention of CVD to know which groups within a population have a high risk for CVD and which risk factors should be targeted with dietary interventions.

In this study of an African population undergoing a nutritional transition in the North West Province of South Africa, we explored the associations between SES (measured by level of urbanisation, education and employment) and CVD risk factors (including diet and nutrient intakes) that were prevalent in 2005 when the baseline PURE data were collected. PURE is an acronym for the 12-year Prospective Urban and Rural Epidemiological study, which is investigating the health transition in urban and rural Africans between 2005 and 2017. The objective of this study was to assess whether social drift in CVD risk has taken place over a period of nine years in Africans of the North West Province, by comparing the findings of the baseline PURE study data to those reported for the THUSA study,^4^ which was collected from 1996 to 1998.

## Methods

This analysis is based on cross-sectional data collected at baseline in 2005 as part of the North West Province, South Africa leg of the international 12-year PURE study. The PURE study is investigating the effects of the health and nutritional transitions, and specifically of NCDs or chronic diseases of lifestyle in urban and rural subjects.

Migration stability was the main selection criterion within the chosen rural and urban communities. Four different areas of residence were used in the subject recruitment for the PURE study. Community A, a rural community, was located 450 km west of Potchefstroom on the highway to Botswana. Community B, a deep rural community 35 km east of A, was only accessible via a gravel road. Communities C and D were urban communities; C was the established Ikageng township, part of the greater Potchefstroom, and D was the informal settlements surrounding community C.

A random household census regarding number of people, their ages and health profiles was done in 6 000 houses (1 500 in each community). Every head of a household gave signed consent to fill in the census questionnaire (see appendix on CVJA website under journal archives). If a person refused or was not at home, the next house was taken and a non-complier questionnaire was filled in. From the data obtained, a paper selection of possible subjects with no reported NCDs, tuberculosis or diagnosed HIV was made.

A total of 2 010 apparently healthy African volunteers, 35 years and older, were then recruited. Participants were fasted (10–12 hours) for the baseline blood sampling and other measurements. Trained field workers assisted in providing information to the participants in their language of choice. Participants received feedback regarding their blood pressure, fasting glucose concentrations and HIV status, and were referred to the nearest clinic or hospital where necessary. Travelling expenses of participants were covered.

Ethical approval was obtained from the Ethics Committee of the North-West University, Potchefstroom, South Africa (ethics number: 04M10) and signed informed consent forms were received from all participants. Subjects were provided with background information on the study and its purpose, and they were informed that participation was voluntary and they could withdraw at any time. Permission to conduct the study was also obtained from the North West Department of Health, tribal chiefs, and the local government authorities of each selected site.

Structured, validated demographic, socio-economic and lifestyle questionnaires were administered by trained field workers during home visits in the language preferred by the participants. The questionnaires used were adapted from those used by all countries participating in the PURE study. A quantitative food frequency questionnaire (QFFQ), validated for this population,^19^ was administered by trained field workers using food models and food photographs of different portion sizes to assess habitual dietary intakes. The food data were converted to nutrient intakes using the South African food composition tables and computer program of the South African Medical Research Council.^20^

Anthropometric measurements were done by trained biokineticists. Height was measured to the nearest 0.5 cm with a stadiometer (Invicta, IP 1465, UK) and weight was determined on a portable electronic scale to the nearest 0.01 kg (Precision Health Scale, A & D company, Japan). All the measurements were done according to the guidelines adopted at the National Institute of Health-sponsored Arlie Conference.^21^ Body circumferences of participants were measured in light underwear with calibrated instruments (Holtain – unstretchable metal tape; John Bull – calipers). Body mass index was calculated by dividing weight in kilograms by height in square metres.

Every participant who signed an informed consent form was tested for HIV infection, but was given the choice of knowing his/her status. Whole blood was used for the determination of HIV status, making use of the First Response (PMC Medical, India) rapid HIV card test. If tested positive, the test was repeated with the Pareeshak (BHAT Bio-tech India) card test. Pre-test counselling was done in groups of 10 persons before the blood sample was taken, and post-test counselling was individualised, according to the protocol of the National Department of Health of South Africa.

A disposable needle was used to draw blood from the antecubital vein in the right arm of the participants. For plasma, each collection tube was filled to its capacity to ensure optimal blood:anticoagulant ratios. The tubes were inverted five times to ensure thorough mixing of the contents of the tube. The tubes were placed in ice boxes after labelling.

A new sterile transfer pipette was used to aliquot blood cell, serum and plasma samples for analysis. Serum was prepared by allowing blood to clot at room temperature for 30 min; it was then centrifuged at 2 000 × *g* for 15 min at 10ºC. Blood was centrifuged within two hours of collection, separated and stored at –70ºC. Plasma samples were collected in ethylenediamine tetra acetic acid (EDTA) tubes, centrifuged at 2 000 × *g* for 15 min at 4ºC and transferred to cryo-tubes for storage at –70ºC.

Both systolic and diastolic blood pressures were obtained using an OMRON automatic digital blood pressure monitor (Omron HEM-757). Subjects did not smoke, eat, exercise or do any intense activities for 30 min before taking the measurements, and they were rested and calmed for 10 min before doing the readings. Blood pressure measurements were performed twice, 5 min apart. Subjects were seated upright and relaxed with the arm supported at heart level and measurements were taken using the brachial artery.

Blood glucose level was measured using Vitros DT6011 Chemistry Analyser, an Ortho-Clinical Diagnostics tool (Rochester, New York, USA). Quantitative determination of total cholesterol (TC), high-density lipoprotein cholesterol (HDL-C), C-reactive protein and triglyceride (TG) levels were done by sequential multiple analyser computer (SMAC) using the Konelab20i^TM^ auto-analyser. Plasma fibrinogen level was measured using a modified Clauss method (Multi fibrin U-test, Dade Behring, Deerfield Illinois, USA) on the Dade Behring BCS coagulation analyser.

## Statistical analysis

The SPSS package (version 17.0, SPSS Inc) was used to analyse the data. Means and 95% confidence intervals (CI) of CVD risk and dietary factors were calculated. Participants of both genders were divided into different groups, according to urbanisation, education and employment levels, and compared. Estimated significant differences between rural and urban participants were determined with analysis of variance using the general linear model (GLM) multivariate procedure. Univariate analysis was used to further explore the influence of education on CVD risk factors and dietary intakes.

Employment was used as a proxy for income, and pairwise comparisons using GLM multivariate procedure was done for comparing the three groups (not answered, employed and not employed). Tests were considered significant at *p* < 0.05.

## Results

The total energy as well as macronutrient intakes and some selected micronutrient levels are shown in Table 1. This shows that mean total energy intakes of urban men and women were significantly higher than those of rural men and women. It can therefore be expected that other nutrient intakes would also be higher in urban subjects because more food was consumed.

**Table 1.** Mean (95% CI) Of Energy And Selected Nutrient Intakes

	*Men*		*Women*	
*Nutrients*	*Urban (n = 399)*	*Rural (n =347)*	*Urban (n = 605)*	*Rural (n = 659)*
Total energy (TE) (kJ)	10054.2 (9681.9–10426.4)^a^	6973.3 (6568.7–7377.8)^a^	9008.3 (8746.0-9269.6)^b^	6107.3 (5854.8–6359.8)^b^
Total protein (g)	74.1 (71.3–76.8)^a^	44.4 (41.4–47.4)^a^	65.54 (63.7–67.4)^b^	39.50 (37.7–41.3)^b^
[% of TE]	[12.5]	[10.8]	[12.4]	[11.0]
Animal protein (g )	33.4 (31.9–35.0)^a^	13.3 (11.6–14.96)^a^	31.2 (30.1–32.2)^b^	12.8 (11.8–13.8)^b^
[% of TE]	[5.6]	[3.2]	[5.9]	[3.6]
Total fat (g)	67.1 (64.3–69.8)^a^	32.4 (29.4–35.3)^a^	67.2 (65.1–69.4)^b^	32.6 (30.5–34.7)^b^
[% of TE]	[25.3]	[17.7]	[28.3]	[20.3]
Saturated fat (g )	16.6 (15.9–17.4)^a^	7.2 (6.3–8.0)^a^	17.2 (16.6–17.8)^b^	7.3 (6.7–7.8)^b^
[% of TE]	[6.3]	[3.9]	[7.3]	[4.5]
Total carbohydrates (g )	334.3 (321.3–347.4)^a^	258.9 (244.7–273.1)^a^	293.7 (284.7–302.8)^b^	235.9 (227.1–244.6)^b^
[% of TE]	[56.5]	[63.1]	[55.4]	[65.7]
Dietary fibre (g)	26.7 (25.6–27.8)^a^	18.5 (17.3–19.7)^a^	22.7 (22.0–23.4)^b^	17.1 (16.4–17.8)^b^
Iron (g)	16.7 (16.0–17.4)^a^	12.4 (11.6–13.1)^a^	14.1 (13.6–14.6)^b^	11.1 (10.6–11.5)^b^
Calcium (g)	452.0 (429.3–474.6)^a^	247.9 (223.2–272.5)^a^	450.9 (434.5–467.3)^b^	211.4 (195.5–227.2)^b^
Vitamin C (g )	42.5 (39.1–45.8)^a^	12.6 (9.0–16.2)^a^	46.9 (44.0–49.8)^b^	15.7 (12.9–18.5)^b^

^a,b^Means with the same symbol differ significantly (*p* < 0.05); significant differences based on GLM, analysis of variance (ANOVA)

However, if differences in the percentages of energy contributed by the different macronutrients are compared, it is clear that not only total amounts of food, but also types of foods and, therefore, dietary patterns differed between urban and rural subjects. In urban men, the contributions of total protein, animal protein, total fat, saturated fat, and total carbohydrates to total energy were 12.5, 5.6, 25.3, 6.3 and 56.5%, respectively. In rural men the corresponding figures were 10.8, 3.2, 17.7, 3.9 and 63.1%. The same pattern was observed when urban and rural women were compared. In rural subjects, animal protein formed about a third of the total protein, while in urban subjects of both genders, about half of the total protein was from animal sources.

Total and saturated fat intakes were twice as high in urban as in rural men and women. Percentage of total energy obtained from fat increased from rural values of 17.7 and 20.3% to urban values of 25.3 and 28.3% for men and women, respectively. This was a major shift from a low-fat, prudent diet followed in the rural areas to a higher-fat, more Westernised type of diet in the urban areas.

Mean intakes of total dietary fibre were significantly higher in urban men and women compared to their rural counterparts. The percentage increases in energy intake from rural to urban men and women were 44 and 48%, while that of dietary fibre were 44 and 33% (percentages not shown in Table 1.).

Mean intakes of selected key micronutrients (iron, calcium and vitamin C) were substantially and significantly higher in urban than rural subjects. For calcium and vitamin C, it was more than double that of rural groups.

In Table 2 the mean levels of CVD risk factors of urban men and women are compared to that of their rural counterparts. Rural women were slightly but significantly younger than urban women. There were no significant differences in total and high-density lipoprotein (HDL) cholesterol levels, but urban women had significantly higher triglyceride levels than rural women. Their mean fasting glucose value was also significantly higher.

**Table 2.** Mean (95% CI) Cardiovascular Disease Risk Factors (Excluding Diet) Of Urban And Rural Subjects

	*Men*		*Women*	
*Risk factor (means and 95% CI)*	*Urban*	*Rural*	*Urban*	*Rural*
Number (*n*)	399	347	605	659
Age (years)	50.4 (49. 3–51.6)	49.4 (48.3 – 50.6)	50.8 (49.8–51.7)^a^	47.5 (46.7–48.3)^a^
High-density lipoprotein cholesterol (mmol/l)	1.62 (1.55–1.69)	1.54 (1.47–1.61)	1.46 (1.40–1.51)	1.51 (1.46–1.56)
Total cholesterol (mmol/l)	4.88 (4.73–5.02)	4.72 (4.58–4.87)	5.20 (5.07–5.32)	5.11 (5.0–5.22)
Fasting glucose (mmol/l)	4.93 (4.76–5.10)	4.81 (4.64–4.99)	5.19 (5.04–5.33)^a^	4.94 (4.80–5.07)^a^
Triglyceride (mmol/l)	1.27 (1.18–1.36)	1.15 (1.06–1.24)	1.47 (1.40–1.53)^a^	1.25 (1.19–1.31)^a^
Systolic blood pressure (mmHg)	137.5 (134.0–140.0)^a^	132.2 (129.6–134.7)^a^	136.7 (134.5–138.8)^b^	127.8 (125.8–129.7)^b^
Diastolic blood pressure (mmHg)	87.9 (86.4–89.5)^a^	84.9 (83.3–86.5)^a^	89.5 (88.2–90.8)^b^	86.4 (85.3–87.5)^b^
Body mass index (kg/m^²^)	20.9 (20.5–21.4)	20.7 (20.2–21.1)	27.8 (27.2–28.5)^a^	25.9 (25.4–26.5)^a^
Fibrinogen (g/l)	3.17 (2.95–3.39)^a^	3.50 (3.28–3.73)^a^	3.74 (3.55–3.93)^b^	4.04 (3.87–4.21)^b^
C-reactive protein (mg/l)	7.87 (6.50–9.23)	8.56 (7.10–10.0)	9.17 (8.18–10.16)	8.09 (7.14–9.04)
Alcohol consumption g/day	17.8 (15.0–20.7)	20.0 (117.0–23.1)	9.0 (7.3–10.5)^b^	6.6 (5.0–8.1)^b^

^a,b^Means with the same symbol differ significantly (*p* < 0.05); significant differences based on GLM, analysis of variance (ANOVA).

Urban men and women had significantly higher blood pressures, and urban women had significantly higher mean BMI than rural women, although the rural women also had a mean BMI in the overweight range. Rural men and women had significantly higher plasma fibrinogen levels. There were no significant differences in C-reactive protein (CRP) between urban and rural groups. Both rural and urban men reported high alcohol intakes (17.8 and 20.0 g/day, respectively). Women took less alcohol, but urban women had significantly higher intakes, namely 9.0 versus 6.6 g/day reported by rural women.

Table 3 shows the mean values of CVD risk factors of men with different reported education levels, while Table 4 gives the same data for women. The values of the 21 men and 37 women who did not answer the questions on education are also shown. In both men and women, the mean age of those with higher education (secondary school and/or additional education) was significantly lower. There were more educated individuals in the urban areas compared to rural areas. However, 52 men and 136 women residing in rural areas also completed secondary schooling and/or additional tertiary education.

**Table 3.** Mean (95% CI) Of Selected Cardiovascular Disease Risk Factors In Men With Different Education Levels

*Risk factor: mean (95% CI)*	*Not answered*	*None*	*Primary*	*Secondary school and higher*
Number of subjects	21	270	297	158
Age in years	48.2 (43.3–53.0)	52.5 (51.2–53.9)	49.4 (48.0–50.7)	45.5 (43.7–47.3)*
Number of urban subjects	9	90	194	106
Number of rural subjects	12	180	103	52
High-density lipoprotein cholesterol (mmol/l)	1.42 (1.11–1.74)	1.68 (1.56–1.73)	1.54 (1.45–1.62)	1.58 (1.46–1.70)
Total cholesterol (mmol/l)	4.62 (3.98–5.25)	4.78 (4.60–4.96)	4.79 (4.62–4.97)	4.89 (4.66–5.13)
Fasting glucose (mmol/l)	4.77 (4.00–5.54)	4.78 (4.56–4.99)	4.93 (4.72–5.15)	4.84 (4.55–5.12)
Triglyceride (mmol/l)	1.15 (0.76–1.54)	1.14 (1.03–1.25)	1.25 (1.14–1.35)	1.27 (1.2–1.41)
C-reactive protein (mg/l)	9.55 (3.73–15.37)	9.30 (7.66–10.94)	8.23 (6.65–9.81)	6.02 (3.88–8.17)
Systolic blood pressure (mmHg)	132.1 (121.2–143.0)	132.9 (29.9–136.0)	136.7 (133.6–139.7)	133.5 (129.4–137.5)
Diastolic blood pressure (mmHg)	86.9 (80.1–93.8)	84.4 (82.4–86.3)	87.4 (85.5–89.3)	86.7 (84.1–89.2)
Body mass index (kg/m^²^)	22.5 (20.6–24.3)	20.0 (19.5–20.5)	21.0 (20.5–21.6)	21.7 (21.0–22.4)*
Fibrinogen (g/l)	3.14 (2.16–4.11)	3.65 (3.78–3.92)	3.17 (2.90–3.44)	3.05 (2.65–3.41)
Total energy (TE) (KJ)	7963 (625–9667)	7626 (7141–8112)	9015 (8555–9474)	9390 (8676–10105)*
Total fat (g) (% of TE)	49.4 (23.6%)	40.7 (20.3%)	52.9 (22.3%)	60.4 (24.4%)*
Total protein (g) (% of TE)	53.9 (11.5%)	51.6 (11.5%)	63.4 (12.0%)	67.9 (12.3%)*
Total carbohydrates (g) (% of TE)	282.3 (60.3%)	273.9 (61.6%)	315.2 (59.4%)	307.7 (55.7%)*
Total fibre (g)	20.0 (15.1–25.0)	20.4 (19.0–21.8)	24.7 (23.3–26.0)	23.4 (21.4–25.4)*
Alcohol consumption (g/day)	12.1 (–0.01–24.3)	17.7 (14.2–21.1)	18.8 (15.5–22.1)	22.0 (17.5–26.5)

*Significant difference across different education levels (*p* < 0.05); significance differences based on GLM, analysis of variance (ANOVA).

Table 3 and 4 further indicate that the serum lipids, glucose and fibrinogen levels did not differ significantly between subjects with different levels of education. In men there were also no differences in blood pressure, but women with higher education had significantly lower systolic and diastolic blood pressures. These women also had lower serum triglyceride levels.

**Table 4.** Mean (95% CI) Of Selected Cardiovascular Disease Risk Factors In Women With Different Education Levels

*Risk factor: mean (95% CI)*	*Not answered*	*None*	*Primary*	*Secondary school and higher*
Number of subjects	37	420	531	276
Number of urban subjects	16	112	337	139
Number of rural subjects	21	308	194	136
Age (years)	47.0 (43.3–50.7)	50.2 (49.1–51.3)	50.7 (49.7–51.7)	44.0 (42.6-45.3)*
High-density lipoprotein cholesterol (mmol/l)	1.36 (1.12–1.59)	1.55 (1.48–1.61)	1.49 (1.43–1.55)	1.43 (1.34–1.52)
Total cholesterol (mmol/l)	5.50 (4.98–6.01)	5.20 (5.05–5.35)	5.19 (5.05–5.33)	4.99 (4.80–5.18)
Fasting glucose (mmol/l)	5.29 (4.73–5.84)	5.04 (4.88–5.21)	4.94 (4.78–5.07)	4.88 (4.67–5.08)
C-reactive protein (mg/l)	10.01 (6.04–14.00)	8.80 (7.61–10.00)	8.67 (7.61–9.73)	8.01 (6.53–9.48)
Triglyceride (mmol/l)	1.38 (1.11–1.65)	1.40 (1.31–1.49)	1.36 (1.28–1.43)	1.22 (1.12–1.32)*
Systolic blood pressure (mmHg)	130.6 (121.9–139.4)	130.4 (127.8–133.0)	134.5 (132.1–136.9)	126.9 (123.7-130.1)*
Diastolic blood pressure (mmHg)	89.5 (84.3–94.7)	86.7 (85.2–88.2)	89.4 (88.0–90.9)	85.7 (83.7–87.6)*
Body mass index (kg/m^²^)	28.6 (26.0–31)	25.2 (24.4–26.0)	27.6 (26.9–28.3)	27.4 (26.5–28.4)*
Fibrinogen (g/l)	4.51 (3.69–5.32)	3.98 (3.74–4.22)	3.96 (3.73–4.18)	3.71 (3.41–4.01)
Total energy (TE) (kJ)	7115 (5985–8245)	6677 (6337–7017)	7997 (7693–8300)	7419 (6960–7877)*
Total fat (g) (% of TE)	45.1 (24.1%)	38.0 (21.6%)	55.4 (26.3%)	50.2 (25.7%)*
Total protein (g) (% of TE)	48.3 (11.5%)	44.6 (11.4%)	56.4 (12.0%)	52.1 (11.9%)*
Total carbohydrates (g) (% of TE)	259.0 (61.9%)	245.3 (62.5%)	274.6 (58.4%)	261.2 (59.8%)
Total fibre (g)	18.1 (15.1–21.1)	18.2 (18.2–19.1)	21.0 (20.2–21.8)	19.3 (18.1–20.5)*
Alcohol consumption (g/day)	4.3 (–2.2–10.9)	10.4 (8.4–12.3)	7.2 (5.5–8.9)	4.9 (2.5–7.4)*

*Significant difference across different education levels population (*p* < 0.05); significant differences based on GLM, analysis of variance (ANOVA).

The BMI of men and women were significantly higher in those subjects who had primary and secondary schooling compared to those without any schooling. The same pattern was observed in the dietary intakes, with sustained increases in energy, fat and protein intake and decreases in carbohydrate intake in men and women with primary education compared to those with no education. This change to a more Westernised diet was sustained in men with secondary education. However, women with secondary education reported a more prudent diet with lower energy, protein and alcohol intakes than those with primary education.

Table 5 compares the CVD risk factors of employed with unemployed men and women. Very few of the rural subjects were employed (15 men and 19 women) while 95 men and 95 women of the urban subjects were employed. Of the total sample of 679 men for whom employment data were available, 84.2% was unemployed, and of the 1 136 women who reported employment status, 90% was unemployed.

**Table 5.** Mean (95% CI) Cardiovascular Disease Risk Factors Of Emloyed And Unemployed Men And Women

	*Men*			*Women*		
*Risk factor: means (95% CI)*	*Not Answered*	*Employed*	*Unemployed*	*Not Answered*	*Employed*	*Unemployed*
Total numbers	49	110	587	128	114	1022
Number of urban subjects	47	95	257	120	9	
Number of rural subjects	2	15	330	8	19	632
Age (years)	52.8 (49.3–56.3)	44.6 (42.4–46.8)^a^	50.4 (49.5–51.4)^b^	50.3 (48.1–52.5)	45.6 (43.4–47.8)^a^	49.1 (48.4–49.9)^b^
High-density lipoprotein cholesterol (mmol/l)	1.46 (1.24–1.69)	1.53 (1.40–1.66)	1.60 (1.54–1.66)	1.19 (1.06–1.32)	1.43 (1.29–1.56)^a^	1.53 (1.49–1.56)^a^
Total cholesterol (mmol/l)	4.62 (4.17–5.07)	4.89 (4.61–5.18)	4.80 (4.68–4.92)	4.93 (4.64–5.210	4.92 (4.62–5.22)	5.20 (5.12–5.30)
Fasting glucose (mmol/l)	5.45 (4.89–6.00)	4.67 (4.31–5.00)^a^	4.84 (4.70–4.99)^a^	5.34 (5.03–5.67)	4.67 (4.35–5.00)^a^	4.96 (4.85–5.07)^a^
C-reactive protein (mg/l)	7.43 (3.04–11.81)	5.81 (3.04–8.58)	8.29 (7.12–9.47)	9.44 (7.04–11.84)	6.05 (3.56–8.54)	8.41 (7.61–9.23)
Triglyceride	1.30 (1.01–1.57)	1.31 (1.13–1.49)	1.18 (1.11–1.26)	1.56 (1.40–1.710	1.16 (0.99–1.31)^a^	1.33 (1.28–1.38)^a^
Systolic blood pressure (mmHg)	138.4 (130.5–146.2)	134.1 (129.2–139.1)	134.2 (132.1–136.3)	133.0 (128.0–138.0)	130.9 (125.7–136.1)	131.1 (129.4–132.8)
Diastolic blood pressure (mmHg)	88.8 (83.8-93.7)	85.8 (82.6-88.9)	86.0 (84.6-87.3)	86.9 (83.9–89.9)	87.8 (84.8-90.9)	87.7 (86.7–88.7)
Body mass index (kg/m^²^)	21.5 (20.1–22.8)	22.1 (21.3–23.0)^a^	20.6 (20.2–20.9)^a^	8.1 (26.6–29.6)	27.9 (26.3–29.4)^b^	26.5 (26.0–27.0)^b^
Fibrinogen (g/l)	2.17 (1.48–2.87)	2.98 (2.55–3.42)^a^	3.47 (3.28–3.65)^a^	3.62 (3.16–4.09)	3.51 (3.03–3.98)^b^	4.01 (3.85–4.16)^b^
Total energy (TE) (kJ)	7626.5 (6248.4–9004.6)	10217.5 (9348.5–11086.4)^a^	8385.7 (8016.6–8754.8)^a^	6318.0 (5612.3–7023.6)	8495.5 (7764.2–9226.9)^b^	7174.6 (6938.3–7410.9)^b^
Total fat (g) (% TE)	51.7 (25.8)	67.9 (25.3)^a^	46.4 (21.0)^a^	45.1 (27.1)	63.9 (28.6)^b^	44.0 (23.3)^b^
Total protein (g) (% TE)	53.5 (11.9)	74.8 (12.4)^a^	57.8 (11.7)^a^	44.9 (12.1)	63.6 (12.7)^b^	48.6 (11.5)^b^
Total carbohydrates (g) (% TE)	245.8 (54.8)	342.7 (57.0)^a^	295.4 (60.0)^a^	214.4 (57.7)	279.9 (56.0)^b^	259.5 (61.5)^b^
Total dietary fibre (g)	19.4 (15.5–23.3)	25.4 (22.9–27.9)^a^	22.3 (21.2–23.3)^a^	15.4 (13.6–17.20)	21.0 (19.1–22.9)^b^	19.4 (18.8–20.0)^b^
Alcohol consumption (g/day)	17.6 (7.6–27.6)	17.7 (11.4–24.0)	20.7 (18.0–23.4)	4.7 (0.5–9.0)	4.0 (–3.9–8.3)	8.2 (6.8–9.6)

^a,b^Means with the same symbol for a specific variable indicate significant differences (*p* < 0.05); significant differences based on GLM analysis of variance (ANOVA).

The employed men and women were significantly younger than the unemployed subjects. The employed men and women had a significantly higher mean BMI and energy intakes but lower HDL cholesterol (women), triglyceride (women), and fibrinogen (men and women) levels. Both the employed and unemployed women had mean BMIs in the overweight range. The diet of the employed showed all the characteristics of the higher-fat, more Westernised diet seen in the urban and more educated groups.

## Discussion

The purpose of this study was to establish the association between SES and CVD risk factors (including dietary intakes) in an African population in the North West province of South Africa. SES was differentiated by use of residential strata (urban or rural), employed or not employed, and education levels (none, primary schooling, or secondary schooling and/or higher education/training). The salient results are summarised briefly and discussed, after which a possible socio-economic drift in the CVD risk factors is critically evaluated.

It should be noted that in the North West province of South Africa, a region taken over by rural-to-urban migration, the majority of those moving are in search of a better life in the cities, where they can possibly get jobs and help those that have stayed behind in the rural setting, usually children, women and the elderly. Using this as the trend in the North West province, one’s setting can be used as a proxy for socio-economic status. Although this might not hold true for everyone, it does for more than 80% of the population, as seen in our 1996 to 1998 results from the THUSA study.^4^ It appears that more processed foods are reaching rural areas, unlike in earlier years.

## Dietary intakes

The increased intakes in total energy, fat and protein, characterised by increases in animal protein and saturated fat, with a concomitant decrease in total carbohydrates taken in during urbanisation confirm the changes in dietary patterns observed during the nutritional transition in other developing countries,^22^ as well as in South Africa.^3^ The observed increase in dietary fibre and micronutrient intake in the urban subjects are probably related to the increased energy and thus food intake. The increases were less than expected for dietary fibre in women, which shows change in the types of foods consumed.

Although the mean increases in selected micronutrients were substantial, they did not reach recommended values, a phenomenon also observed in other urban black South Africans.^23^ The observation that intakes of macronutrients by the more highly educated women compared to those with little or no education were actually more prudent (lower in energy and fat) suggests that the nutritional transition may have reached a point in these women where healthier diets are now being followed.

## CVD risk factors

The only significant difference between rural and urban subjects regarding serum lipid levels was the increased triglyceride levels in urban women. The same group was significantly older than the rural women, had a higher fasting mean glucose level, and higher BMI. It is therefore possible that the higher triglyceride and glucose levels in urban women could be related to the older age and higher BMI, both known to influence these variables.^17^

Both urban men and women had higher mean blood pressures, while the rural subjects had higher mean plasma fibrinogen levels. Plasma fibrinogen is accepted as a risk factor for CVD.^24^ In addition, fibrinogen is an acute-phase reactant.^25^ The higher fibrinogen levels in the rural subjects could therefore also reflect chronic (perhaps low-grade) infection despite the fact that apparently healthy subjects were recruited for the PURE study. However, the mean values of the highly sensitive C-reactive protein did not differ significantly between urban and rural men and women. In the THUSA study,^26^ urban subjects tended to have higher plasma fibrinogen levels, although values were also raised in rural subjects, especially in those living on commercial farms.

## Effects of educational level

The salient observations regarding the effects of educational level on CVD risk factors were that with increased education there were increases in BMI and in energy and macronutrient intakes of both men and women when those with primary education were compared to those with no education. This change to a Westernised diet was sustained in men with secondary education but not in women.

The other CVD risk factors did not show significant differences in uneducated and educated subjects, except for lower serum triglyceride levels and blood pressures in the more educated women. However, these women were also slightly but significantly younger than the uneducated women and it seems that they followed a more prudent diet than women with only primary schooling. The diet of the educated subjects resembled that of the urban subjects, indicative of the changes observed in the nutritional transition, with indications that in the highly educated women, energy and macronutrient intakes were changing back to more prudent intakes.

## Effects of employment

Of the 1 833 subjects for whom data on employment were available, 84.2% of the men and 90.0% of the women were unemployed, which may indicate a bias in the sample selection. This bias could be the result of recruiting volunteers who were available for a very long weekday of measurements, for which employed people would have to take leave. The official figure of the unemployment rate in South Africa between 2000 and 2006 averaged at 26.38%, with the highest in March 2003, at 31%.^27^

However, the PURE questionnaires used to assess employment status have a category described as homemaker, which is interpreted as unemployment. It is possible that employed domestic workers, especially in the urban areas, indicated this category, which may be partially responsible for the high unemployment figure. The employed subjects were significantly younger than the unemployed. This may explain why, despite the higher fat intake and more Westernised diet, the employed men and women had significantly lower fasting glucose and triglyceride (women) levels.

## Is there a social drift in CVD risk factors in this population?

To evaluate this question, one should consider the limitations of the study. Firstly, as already mentioned, the reported employment rates may suggest that the sample was biased. However, potential bias would be similar for all four sites where subjects were recruited. A second limitation was the absence of reliable data on personal income. In many African families, household income from different sources is shared by a varying number of extended family members.^28^

As proxy for socio-economic status, educational level, employment and residing in urban or rural areas were used. The data showed that many subjects with higher education were employed in the rural areas. None of these three variables is an ideal, independent indicator of SES, but it was assumed that each could be used to distinguish between higher and lower socio-economic groups. Nevertheless, the data should be interpreted with care.

Patterns of dietary intakes and risk factors for CVD emerged, regardless of which indicator of socio-economic status was used, with agreement between results of being urban, highly educated and employed. However, as indicated above, there were also some exceptions, which could be explained.

The analysis of the relationships between socio-economic position and CVD risk factors in the participants of the THUSA study4 showed that nine years earlier, most (but not all) of the CVD risk factors were significantly higher in the subjects from the higher socio-economic group. The major difference between the THUSA study results and the PURE study results reported here was that total serum cholesterol levels did not differ between the higher and lower socio-economic groups, and that increased plasma fibrinogen levels were higher in subjects from the lower socio-economic groups in the PURE subjects.

Although blood pressures were higher in urban subjects, in those groups with higher educational levels or employed, blood pressures did not differ significantly or were even lower than in those with lower educational levels or unemployed. These results suggest a drift of CVD risk factors (lipids and fibrinogen) from participants with high SES to those with lower SES.

The changes in dietary intakes are intriguing. The typical increases in energy and fat intake associated with urbanisation were seen in the PURE subjects (Table 1). However, in women with the highest educational level, there were significant decreases in total energy and fat intake, suggesting that these women were now following a more prudent diet. As for urban participants, men with higher educational levels and employed men and women still had higher energy and fat intakes, reflected in higher BMIs and serum triglyceride levels. This raises the question whether diet was in anyway responsible for the drift of the CVD risk factors.

It seems that the contribution of low intake of macronutrients should also be considered. It has been mentioned that although micronutrient intake of the urban subjects increased, recommended values were not reached. James and co-workers^26^ showed in the THUSA study that low micronutrient status was associated with increased plasma fibrinogen levels. Also, it is known that several antioxidant micronutrients protect against CVD and other NCDs.^17^ It is therefore reasonable to suggest that not only a prudent diet regarding macronutrients, but also an adequate diet regarding micronutrients is a prerequisite for dietary protection against CVD.

The intakes of dietary fibre in all groups were low (see Table 1). High intake of dietary fibre (from whole grains, fruit and vegetables) is known to protect against CVD.^29^

Physical activity is a key determinant of CVD risk and should always be taken into account. In this study it was not reported, as the focus of this study was to observe whether a shift had occurred prior to the results we published on the THUSA study.^4^

Different demographics, including racial and religious heterogeneity of populations, may have an impact on dietary patterns. Since this epidemiological survey was composed of 2 010 black Africans undergoing transition and predominantly Christian, we state that religion did confound our results or act as modifier effectors to these associations.

These are baseline results from a prospective epidemiological study that is on-going. Therefore findings reported here give us an insight into the associations between SES and specific CVD risk factors. These observations have limitations in that they were carried out at one point in time and give no indication of the sequence of events – whether exposure occurred before, during or after the onset of the outcome. This being so, it is impossible to infer causality.

After subsequent follow ups (prospective), one of the advantages of such a study is that it can help determine and observe risk factors in those participants previously free of risk factors. Because the studies are longitudinal over time, and the collection of results is at regular intervals, recall error is minimised.

## Conclusions

South Africa is undergoing political changes which have led to rapid economic development and urbanisation of its African people. This has led to an increase in rural–urban migration, thus exposing the ‘once-protected’ black rural population to more Westernised dietary habits. These ultimately lead to the observed increase in incidence of NCDs in this population.^30^

The results of this study showed that urban, educated and employed subjects had high levels of several dietary and biochemical risk factors for CVD. However, the results also indicated that many people living in the rural areas of the North West province and those who had lower educational levels and were unemployed also had an increased risk of CVD despite still following a prudent but micronutrient-deficient diet.

It was therefore concluded that the burden of CVD is shifting from the more affluent groups with higher SES to the poor. At this time, it seems that many of the risk factors of CVD are prevalent in all SES groups of black South Africans.

It is therefore recommended that intervention programmes to prevent CVD and other NCDs should be targeted at all SES groups. Furthermore, efforts to improve dietary and nutrient intakes should not only focus on steering the nutritional transition into consumption of a more prudent, low-energy, low-fat diet, but should also ensure that sufficient micronutrients are taken in by emphasising the importance of a varied diet with sufficient amounts of nutrient-dense foods.
